# Updated Estimate of the Number of Extreme Risk Protection Orders Needed to Prevent 1 Suicide

**DOI:** 10.1001/jamanetworkopen.2024.14864

**Published:** 2024-06-12

**Authors:** Matthew Miller, Yifan Zhang, David M. Studdert, Sonja Swanson

**Affiliations:** 1 Department of Health Sciences, Bouvé College of Health Sciences, Northeastern University, Boston, Massachusetts; 2 Department of Health Policy, Stanford School of Medicine, Stanford, California; 3 Stanford Law School, Stanford, California; 4 Department of Epidemiology, University of Pittsburgh School of Public Health, Pittsburgh, Pennsylvania

## Abstract

**Importance:**

Extreme risk protection orders (ERPOs)—also known as red flag, risk warrant, and gun violence restraining orders—authorize law enforcement, family members, and sometimes others to petition a court to remove firearms from and prevent the acquisition of new firearms by a person judged to pose an immediate danger to themselves or others. Previous estimates suggest that 1 suicide is prevented for every 10 ERPOs issued, a number needed to treat that depends critically on the counterfactual estimate of the proportion of suicidal acts by ERPO respondents that would have involved firearms in the absence of ERPOs.

**Objective:**

To empirically inform updated estimates of the number of ERPOs needed to prevent 1 suicide.

**Design, Setting, and Participants:**

This cohort study used data from California for method-specific suicides by handgun ownership (October 18, 2004, to December 31, 2015). Handgun-owning suicide decedents in California were identified using individual-level registry data about lawful handgun ownership linked to cause-specific mortality for a cohort of more than 25 million adults. The study also used data from Connecticut for method-specific suicides among ERPO respondents who died by suicide, extracted from published data (October 1999 to June 2013). Data analysis was performed in December 2023.

**Exposure:**

Handgun ownership.

**Main Outcomes and Measures:**

The primary outcomes were the number and distribution of suicidal acts by handgun owners in California, estimated using method-specific suicide mortality data and published case fatality ratios, and the counterfactual number and distribution of suicidal acts and deaths among ERPO respondents in Connecticut had no ERPOs been issued.

**Results:**

A total of 1216 handgun owners (mean [SD] age, 50 [18] years; 1019 male [83.8%]) died by suicide during the study period. Among male handgun owners in California, 28% of suicidal acts involved firearms, 54% involved drug poisoning, 9% involved cutting or piercing, 3% involved hanging or suffocation, 2% involved poisoning with solids and/or liquids, and the remaining 4% involved other methods. Assuming this distribution approximates the counterfactual distribution among ERPO respondents in Connecticut in the absence of ERPOs, 1 suicide death was prevented for every 22 ERPOs issued.

**Conclusions and Relevance:**

The estimates produced by this cohort study of California handgun owners suggest that ERPOs can play an important role in averting deaths among high-risk individuals.

## Key Points

**Question:**

How effective are extreme risk protection orders (ERPOs) in preventing suicide for the people subject to these emergency measures?

**Findings:**

This cohort study, which combines information about the suicide methods used by handgun-owning suicide decedents in California, the lethality of different suicide methods, and the distribution of suicide deaths among ERPO respondents in Connecticut, found that 1 suicide death was prevented for every 22 ERPOs issued.

**Meaning:**

These findings suggest that ERPOs can play an important role in averting deaths among high-risk individuals.

## Teaser

This cohort study uses data for handgun owners in California who died by suicide and for extreme risk protection order (ERPO) respondents in Connecticut who died by suicide to estimate of the number of ERPOs needed to prevent 1 suicide.

## Introduction

Extreme risk protection orders (ERPOs) are civil court orders designed to temporarily prevent a person from purchasing or possessing firearms and ammunition.^1^ These laws—also known as red flag, risk warrant, and gun violence restraining orders—authorize law enforcement, family members, and sometimes others (eg, health care professionals or coworkers) to petition a court to remove firearms from and prevent the acquisition of new firearms by a person judged to pose an immediate danger to themselves or others. By March 2024, 21 states and the District of Columbia had enacted such laws.^1^ As with many firearm laws, most US adults do not know whether their state has ERPOs or what they entail.^2^ However, once informed, a majority of the public express support for ERPO laws.^3^

Evidence about whether ERPO laws prevent suicide at the population level is mixed, with some studies finding no effect and others finding modest reductions in rates of firearm suicide and overall suicide.^4,5,6^ The most recent of these population-level studies^5^ called for additional investigation to estimate individual-level benefits of ERPOs, noting specifically that strong effects on those directly affected by these orders could be present even if the population-level effect is minimal.

To our knowledge, no study to date has directly compared the suicide rate among ERPO respondents (ie, persons who received an ERPO) to the rate among a comparable population who are not ERPO respondents. However, 2 prominent studies, both by Swanson and colleagues,^7,8^ used indirect methods to estimate the number of ERPOs issued to prevent 1 suicide death (ie, a number needed to treat [NNT]). Those studies focused on ERPO respondents who died by suicide (21 in Connecticut, 1999-2013; 14 in Indiana, 2006-2013). The overlapping conclusion of both studies was that 10 ERPOs need to be issued to prevent 1 suicide death, or, as the abstract of the later study states, “one life was saved for every 10 gun-removal actions, similar to results of a previous study in Connecticut.”^8^ In the Connecticut study,^7^ Swanson et al also present what they considered to be an overly conservative estimate of approximately 20 suicides averted for every ERPO issued to Connecticut residents.

The approach Swanson and colleagues^7^ used involved 3 steps, which we describe here to illustrate how data from the Connecticut study produced an NNT of 10. First, using information about suicide deaths by method among all ERPO respondents, Swanson et al^7^ derived an estimate of the number of suicidal acts (ie, suicides plus nonfatal suicide attempts) made by all ERPO respondents by summing across all methods the number of suicidal acts by each method (the latter was derived by dividing the number of method-specific suicide deaths by the corresponding method-specific case fatality ratio [CFR] for male individuals). Male CFRs were used because 86% of suicides were among male individuals, as were 92% of suicides among ERPO respondents. Second, Swanson et al^7^ assumed that the same number of suicidal acts would have occurred had no ERPOs been issued. Third, to calculate the counterfactual number of suicide deaths, method-specific CFRs were applied to a conjectured counterfactual method distribution among the estimated total number of suicidal acts. In arriving at an estimated NNT of 10, Swanson et al^7^ assumed that in the absence of ERPOs, 70% of suicidal acts by ERPO respondents would have involved firearms. For the remaining 30%, they assumed a distribution of nonfirearm methods that mirrored the (CFR-derived) nonfirearm method distribution produced in the first step. Given the method-specific CFRs for each method, this distribution corresponded to an aggregate counterfactual suicide death in the Connecticut study^7^ of 93, which was 72 suicides in excess of the 21 suicides that had, in fact, occurred among the 762 ERPO respondents. The difference between the counterfactual and the observed suicide death counts is the basis for the widely cited estimate that 1 suicide death was prevented for approximately every 10 ERPOs served—that is, NNT = 1 / [(93 − 21) / 762] = 10.6—which is equivalent to an estimated causal risk difference of 9.4 percentage points (ie, 72 / 762).^1,9^

The NNT that Swanson et al chose to report as their only estimate in the Indiana study^8^ and to exemplify in the Connecticut study^7^ was an NNT of 10. In the Connecticut study,^7^ the NNT of 10 depended critically on the conjecture that 70% of suicidal acts by ERPO respondents would have involved firearms in the absence of ERPOs. Had the Indiana study^8^ used the 70% statistic, rather than 39% as they did in that study, their NNT would have been approximately 5. The current study updates the NNT estimates from Swanson and colleagues^7,8^ by incorporating individual-level data about handgun ownership among suicide decedents in California to inform the counterfactual distribution of methods that would have been used in suicide acts by ERPO respondents in Connecticut under the counterfactual scenario that the intervention had not occurred and their firearms had not been removed. Specifically, we used individual-level data about lawful handgun ownership in California linked to cause-specific mortality for a cohort of more than 25 million adults tracked from October 18, 2004, through December 31, 2015, to identify the method-specific distribution of suicide deaths for handgun owners. Next, we derived an estimate of the aggregate number and distribution of suicidal acts by handgun owners in our California cohort (based on the observed method distribution among our suicide decedents and published sex-specific and method-specific CFRs). Finally, following the approach used by Swanson et al,^7,8^ but substituting our method-specific distributional estimate of suicidal acts for the investigators’ conjectured counterfactual distribution, we provide an empirically updated estimate of the outcomes of ERPOs for the ERPO respondents in the Connecticut study,^7^ expressed as the estimated number of ERPOs issued for every suicide death prevented.

## Methods

This cohort study follows the Strengthening the Reporting of Observational Studies in Epidemiology (STROBE) reporting guidelines for observational studies and was approved by the institutional review board at Stanford University.^10^ Informed consent was not obtained because all individuals were deceased.

### Firearm Ownership Data in the LongSHOT Cohort

Virtually all lawful transfers of firearms in California, including transfers between private parties, gifts, and loans, must be transacted through a licensed firearms dealer. Dealers relay details of the transfers and transferees electronically to the California Department of Justice, where the information is archived in the Dealer Record of Sale (DROS) database. People who move to California with firearms are required to report or transfer their weapons within 60 days after arrival, and these reports are also entered into the database. Although this legal regimen has governed handgun transfers for decades, transfers of long guns (rifles and shotguns) were not routinely archived until January 1, 2014.^11^

The cohort construction is described in detail elsewhere.^11,12,13^ Briefly, the LongSHOT cohort was formed by linking information on handgun transactions and all-cause mortality among adults in California to a series of historical extracts of the California Statewide Voter Registration Database (SVRD). The SVRD enumerates all registered voters in the state. Because the SVRD must be kept up to date—with new registrations, deregistrations, and changes of names and addresses—extracts present a sample of adults known to be alive and residing in California on the extract date. Using records of the 9.1 million handgun and long gun transfers recorded in the DROS database over a 32-year period (January 1, 1985, through December 31, 2016), we constructed 3 additional variables. First, to identify cohort members who already owned a handgun at the beginning of the study period, we linked data on handgun transfers in the 19.8 years leading up to the study period (2004-2016). Second, we created a time-varying variable that indicated the cumulative number of handguns owned (based on acquisitions and deacquisitions) and used it to identify divestments—that is, transfers of the last known handgun a cohort member owned. The age and sex of cohort members were derived from the SVRD. Data on race and ethnicity were derived from the SVRD and are categorized as Asian, Black, Hispanic, White, and other (any other US Census category not otherwise specified). Data on race and ethnicity were included in this study to provide context about gun ownership in California. Missing values for sex and race were imputed with use of validated methods described in detail elsewhere.^11,14,15^ Because California enacted its first ERPO in 2016, we restrict our analytic sample to data from the LongSHOT cohort through December 31, 2015.

### California Mortality Data

Causes of death were coded according to the *International Statistical Classification of Diseases and Related Health Problems, Tenth Revision*, in which suicides are specified according to method (codes X60-X84). Mortality data were linked to handgun exposure status using DROS data that indicated which cohort members acquired handguns and the dates of acquisition.

### Statistical Analysis

#### Estimation of Suicidal Acts by Handgun Ownership Status and Sex in LongSHOT

The number of suicidal acts (fatal plus nonfatal) by method was estimated by dividing the number of method-specific suicide fatalities by their corresponding method-specific CFRs.^7,16^ The CFR we used was based on all self-inflicted deliberate self-harm episodes that resulted in an emergency department visit (Swanson et al,^7^ by contrast, used an inpatient database).^17^

#### Estimation of Factual and Counterfactual Suicidal Acts and Suicide Deaths Among ERPO Respondents in Connecticut

Borrowing from the study by Swanson et al,^7^ we estimated the number of suicidal acts made by the 762 ERPO respondents in Connecticut by dividing the number of method-specific suicide deaths that occurred by the corresponding method-specific CFR for male individuals and aggregating over all methods. We assumed that the same number of suicidal acts would have occurred had no ERPOs been issued and distributed these acts by method to match the method-specific distribution of suicidal acts derived above for male handgun owners in LongSHOT. Finally, we derived the counterfactual number of suicide deaths among ERPO respondents by multiplying the method-specific CFR for male individuals by the counterfactual method-specific number of suicidal acts, and summing across methods. Data analysis was performed in December 2023 using R statistical software version 4.1.1 (R Project for Statistical Computing) and Stata statistical software version 14.1 (StataCorp).

## Results

### Characteristics of Handgun Owners and Nonowners Who Died by Suicide in LongSHOT

A total of 1216 handgun owners and 15 164 nonowners died by suicide during the study period. Handgun-owning suicide decedents were younger than nonowner suicide decedents at the time of their death (mean [SD] age, 50 [18] years vs 53 [18] years) and were more likely to be male (1019 individuals [83.8%] vs 10 451 individuals [68.9%]) and White (1016 individuals [8.3.6%] vs 12 105 individuals [7.98%]) (Table 1). Among handgun owners, 53 (4.4%) were Asian, 55 (4.5%) were Black, and 92 (7.6%) were Hispanic. Among nonowners, 806 (5.3%) were Asian, 656 (4.3%) were Black, and 1597 (10.5%) were Hispanic.

**Table 1.** Characteristics of Individuals Who Died by Suicide According to Handgun Ownership Status

Characteristic	Individuals, No. (%)	
	Owners (n = 1216)	Nonowners (n = 15 164)
Sex		
Male	1019 (83.8)	10 451 (68.9)
Female	197 (16.2)	4713 (31.1)
Age, y		
Mean (SD)	50 (18)	53 (18)
Median (range)	51 (21-95)	52 (21-102)
Race or ethnicity		
Asian	53 (4.4)	806 (5.3)
Black	55 (4.5)	656 (4.3)
Hispanic	92 (7.6)	1597 (10.5)
White	1016 (83.6)	12 105 (79.8)
Other^a^	0	0
Residential location		
Urban	1064 (87.5)	13 014 (85.8)
Suburban	97 (8)	1398 (9.2)
Large rural town	32 (2.6)	456 (3)
Small rural town	23 (1.9)	296 (2)
Missing	0	0

^a^ Race and ethnicity for the cohort were imputed using the method developed by Imai et al^15^ for imputing these variables in voter file records. Other refers to all categories reported in US Census data, except the 4 categories specified.

### Method-Specific Suicide Mortality Distribution in LongSHOT by Ownership and Sex

Of the 1216 suicides among handgun owners, 88% involved firearms among male and female owners, respectively (Table 2). Suicide by hanging and by drug poisoning comprised most of the remaining suicides among male and female handgun owners. One-third (33%) of all suicides by nonowners involved firearms (40% among male nonowners and 17% among female nonowners).

**Table 2.** Method-Specific Suicide Mortality Distribution by Handgun Ownership and Sex in the LongSHOT Cohort

Method	Suicides, %					
	Handgun owners (n = 1216)	Nonowners (n = 15 164)	Handgun owners		Nonowners	
			Male (n = 1019)	Female (n = 197)	Male (n = 10 451)	Female (n = 4713)
Firearm	88	33	88	88	40	17
Poisoning with drugs	5	24	5	7	15	44
Poisoning with other solid and/or liquid substances	<1	<1	<1	<1	<1	<1
Poisoning with gases and vapors	<1	3	<1	<1	3	3
Hanging	6	29	6	6	30	27
Drowning	<1	2	<1	<1	<1	2
Cutting	<1	3	<1	<1	3	2
Jumping from a height	<1	5	<1	<1	5	5
Jumping or lying before a moving object	<1	2	<1	<1	2	2
Other or unspecified means	<1	2	<1	<1	2	2

### Estimated Number and Method Distribution of Suicidal Acts in LongSHOT by Ownership and Sex

Of the 4871 suicidal acts we estimated had occurred among all handgun owners, we estimate 3531 were made by male handgun owners, of which 28% involved firearms, 54% drug poisoning, 3% hanging or suffocation, 2% poisoning with solids or liquids, and 9% cutting or piercing (Table 3). Among male suicide attempters who did not own handguns, we estimated that approximately 4% of suicidal acts involved firearms.

**Table 3.** Estimated Method Distribution of Suicidal Acts by Sex in the LongSHOT Cohort

Method	Suicidal acts, %					
	Handgun owners (n = 4871)	Nonowners (n = 323 152)	Handgun owners		Nonowners	
			Male (n = 3531)	Female (n = 1340)	Male (n = 116 950)	Female (n = 206 202)
Firearm	25	2	28	15	4	<1
Poisoning with drugs	57	59	54	65	53	62
Poisoning with other solid/liquid substances	4	4	2	11	5	4
Poisoning with gases and vapors	<1	<1	<1	<1	<1	<1
Hanging	3	3	3	2	5	<1
Drowning	<1	<1	<1	<1	<1	<1
Cutting	6	25	9	<1	20	27
Jumping from a height	<1	<1	<1	<1	2	<1
Jumping or lying before a moving object	<1	<1	<1	<1	<1	<1
Other or unspecified means	3	6	<1	6	10	4

### Estimated Number of Suicide Deaths Prevented by ERPOs Among ERPO Respondents in Connecticut

We estimate that the 21 suicide deaths among the 762 ERPO respondents in Connecticut resulted from a total of 187 suicidal acts, based on the method-specific distribution of suicide deaths reported by Swanson et al^7^ and the corresponding method-specific CFRs for male individuals published by Conner et al^17^ (Table 4). Applying the method distribution derived in Table 3 for handgun-owning male individuals in LongSHOT to these 187 suicidal acts produced a counterfactual estimate that 52 suicidal acts would have involved firearms had no ERPOS had been issued (ie, 28% of 187) (Table 4). Of these 52 suicidal acts, we estimate that 47 would have been fatal. The counterfactual number of nonfirearm suicides was derived analogously for the remaining 135 suicidal acts involving nonfirearm methods, yielding 8 nonfirearm suicide deaths. Thus, our counterfactual suicide deaths consisted of a total of 55 suicides. Comparing this estimate with the 21 that had, in fact, occurred, yields an estimated 34 suicides that were prevented among the 762 ERPO respondents, or 1 suicide for every 22 ERPOs issued over the study period (NNT = 22.4).

**Table 4.** Step-by-Step Derivation of the Estimated Number of ERPOs Issued for Every Suicide Death Prevented in Connecticut, 1999-2013

Method of deliberate self-harm	Suicide deaths, No.^a^	Method-specific CFR for male individuals^b^	Estimated No. of suicidal acts among ERPO respondents^a^^,^^c^	Method-specific attempt distribution among handgun-owning male individuals in LongShot, %^d^	Counterfactual distribution of suicidal acts assuming the method-specific attempt distribution among handgun-owning male individuals in LongShot	Counterfactual No. of suicide deaths assuming the method-specific CFR for male individuals	No. of ERPOs issued for every suicide death prevented
Firearm	6	0.904	6.6	28	52.4	47.3	NA
Poisoning with drugs	2	0.025	80.0	54	101.0	2.5	NA
Poisoning with other solid or liquid substances	0	0.016	0.0	2	3.7	0.1	NA
Poisoning with gases and vapors	2	0.342	5.9	1	1.9	0.6	NA
Hanging	10	0.559	17.9	3	5.6	3.1	NA
Drowning	0	0.62	0.0	0	0	0	NA
Cutting	1	0.013	76.9	9	16.8	0.2	NA
Jumping from a height	0	0.306	0.0	1	1.9	0.6	NA
Jumping or lying before a moving object	0	0.307	0.0	1	1.9	0.6	NA
Other or unspecified means	0	0.02	0.0	1	1.9	0.04	NA
All methods combined	21	0.147	187.0	0	187	55.1	22.4

Abbreviations: CFR, case fatality ratio; ERPO, extreme risk protection order; NA, not applicable.

^a^ Data in this column are from Swanson et al.^7^

^b^ Data in this column are from Conner et al.^17^

^c^ The estimated number of method-specific suicidal acts among the 21 total suicide deaths among ERPO respondents in Swanson et al^7^ (column 4) are derived by dividing the number of suicide deaths (column 2) by the method-specific CFR in column 3. Given that the estimated method-specific number of suicidal acts sums to 187 suicidal acts, applying the method-specific suicide attempt distribution taken from Table 3 (column 5), these 187 suicides attempts are distributed across rows of suicide methods (column 6). The counterfactual number of suicide deaths by method is calculated by dividing the number of method-specific suicidal acts (column 6) by the corresponding CFR (column 3). Finally, the counterfactual total number of suicides is calculated by summing across all methods.

^d^ Data in this column are from Table 3.

## Discussion

In this cohort study, by combining the observed distribution of suicide methods used by handgun-owning suicide decedents in our California cohort with published estimates of the lethality of different suicide methods, we derived an estimate of the number of suicidal acts made by male handgun owners in our sample and the distribution of methods used in these acts (eg, 28% of suicidal acts involved firearms). We used this empirically derived distribution as the estimated distribution of suicidal acts that would have occurred among ERPO respondents in the Connecticut study^7^ had they, counter to fact, not been subject to ERPOs. Following the general approach of Swanson and colleagues^7^ otherwise, we estimated that 1 suicide death was prevented for every 22 ERPOs issued under Connecticut’s risk warrant law between October 1999 and June 2013.

The primary reason for the discrepancy between the NNT of 10 found in the Connecticut study by Swanson et al^7^ and our NNT of 22 is that Swanson and colleagues assumed a counterfactual distribution of suicidal acts in which 70% of ERPO respondents used firearms, whereas our counterfactual estimate was 28%. It is not clear why the investigators settled on 70% to illustrate their approach and as the proportion they highlight in their results. This proportion is much larger than the data-derived estimate of 39% they present as the proportion of suicidal acts involving firearms for members of households with guns (derived from state-level regressions involving the proportion of male suicides that are firearm suicides and survey-based estimates of household firearm prevalence, the latter using the 2013 YouGov survey).^7,18^ Although Swanson et al^7^ did not comment on the impact of using YouGov survey data in these state-level regressions (YouGov is not representative at the state level), they explicitly considered the 39% statistic too conservative because it did not account for a postulated excess risk of gun suicide by ERPO respondents, presumably relative to the propensity to use firearms in suicidal acts among male suicide attempters who live in household with firearms. By contrast, using ecologic data at the national level, and assuming that the relative risk of dying by suicide is 3 times higher for people living in a household with vs without firearms, Hemenway^16^ estimated that firearms are involved in approximately 20% of suicidal acts by members of households with guns.

### Limitations

Several limitations should be kept in mind when interpreting our findings. First, the absolute value of our primary outcome (NNT = 22) is only one of many considerations pertinent to evaluating the potential risks and benefits of ERPO laws. Other potential benefits, such as averting homicides and mass shootings and alleviating stress among family and friends of an individual in crisis, need to be weighed against possible harms, such as intrusions into liberty interests and the likelihood that ERPOs would precipitate harms when law enforcement officers attempt to remove firearms.^19,20^

Second, the NNT we derived does not address how the effect of ERPOs might vary depending on how, where, and why they are implemented. For example, the number of suicides that ERPOs can prevent depends on the underlying risk of the people targeted (eg, some states predominantly target gun owners at risk of suicide, whereas other states target those at risk of harming others), the number of people targeted, and on other contextual factors plausibly related to how well ERPOs reduce access to firearms (eg, whether background checks are required for private firearm sales and the evidentiary standards of a state’s ERPO law). Although heterogeneity is likely, and argues against interpreting our NNT estimate (or the NNT estimates of Swanson and colleagues^7^) as the NNT for ERPOs generally, our findings nonetheless provide an empirically anchored estimate for a small group of gun owners at substantially elevated risk of suicide.^21,22^

Third, the findings from our study rest on 3 assumptions we made to account for events we could not observe directly. First, following Swanson et al,^7^ we assumed that we could estimate the number of suicidal acts (made by ERPO respondents in Connecticut and by handgun owners in LongSHOT) by dividing the observed number of method-specific suicide deaths by published method-specific CFRs. Second, following Swanson et al,^7^ we assumed that ERPO-related gun removal affected the distribution of methods used by ERPO respondents but not the number of suicidal acts. Third, we assumed that the method-specific distribution of suicidal acts we derived for registered voters in California who lawfully owned handguns was a reasonable approximation of the counterfactual method distribution that would have been used by ERPO respondents in Connecticut in the absence of ERPOs.

## Conclusions

ERPO laws attempt to target a relatively small population of people who appear to pose a clear and present danger to themselves or others but who are not otherwise restricted from purchasing or possessing firearms and ammunition. The findings of this study suggest that Connecticut’s use of ERPOs, from 1999 to 2013, prevented 1 suicide for every 22 ERPOs issued. As policymakers attempt to learn how to use ERPOs most effectively and equitably, the heterogeneous US landscape presents an opportunity for future studies to identify and learn more about the effect of different ERPO implementation approaches, which, in turn, could help guide policy and legislative decisions.

## Notes

Supplement.
Data Sharing Statement
