# Supplementary material for: Updated Estimate of the Number of Extreme Risk Protection Orders Needed to Prevent 1 Suicide
Source: JAMA Netw Open. 2024 Jun 12;7(6):e2414864. doi: 10.1001/jamanetworkopen.2024.14864 (PMC11170301; doi:10.1001/jamanetworkopen.2024.14864)
Supplement: Supplement. — Data Sharing Statement [file jamanetwopen-e2414864-s001.pdf]

Data Sharing Statement

Miller. Updated Estimate of the Number of Extreme Risk Protection Orders Needed to Prevent 1 Suicide. *JAMA Netw Open*. Published June 12, 2024.
doi:10.1001/jamanetworkopen.2024.14864

Data
Data available: No

Additional Information
Explanation for why data not available: The paper's tables contain all the relevant aggregate data. Our MOU with the Department of Justice does not allow sharing individual-level data from the firearms registry.
